# The AMC Linear Disability Score (ALDS): a cross-sectional study with a new generic instrument to measure disability applied to patients with peripheral arterial disease

**DOI:** 10.1186/1477-7525-7-88

**Published:** 2009-10-12

**Authors:** Rosemarie Met, Jim A Reekers, Mark JW Koelemay, Dink A Legemate, Rob J de Haan

**Affiliations:** 1 Department of Radiology, Academic Medical Center, Meibergdreef 9, 1105 AZ, Amsterdam, the Netherlands; 2 Department of Vascular Surgery, Academic Medical Center, Meibergdreef 9, 1105 AZ, Amsterdam, the Netherlands; 3 Department of Clinical Epidemiology and Biostatistics, Academic Medical Center, Meibergdreef 9, 1105 AZ, Amsterdam, the Netherlands

## Abstract

**Background:**

The AMC Linear Disability Score (ALDS) is a calibrated generic itembank to measure the level of physical disability in patients with chronic diseases. The ALDS has already been validated in different patient populations suffering from chronic diseases. The aim of this study was to assess the clinimetric properties of the ALDS in patients with peripheral arterial disease.

**Methods:**

Patients with intermittent claudication (IC) and critical limb ischemia (CLI) presenting from January 2007 through November 2007 were included. Risk factors for atherosclerosis, ankle/brachial index and toe pressure, the Vascular Quality of Life Questionnaire (VascuQol), and the ALDS were recorded. To compare ALDS and VascuQol scores between the two patient groups, an unpaired *t*-test was used. Correlations were determined between VascuQol, ALDS and pressure measurements.

**Results:**

Sixty-two patients were included (44 male, mean ± sd age was 68 ± 11 years) with IC (n = 26) and CLI (n = 36). The average ALDS was significantly higher in patients with IC (80, ± 10) compared to patients with CLI (64, ± 18). Internal reliability consistency of the ALDS expressed as Cronbach's α coefficient was excellent (α > 0.90). There was a strong convergent correlation between the ALDS and the disability related Activity domain of the VascuQol (*r *= 0.64).

**Conclusion:**

The ALDS is a promising clinimetric instrument to measure disability in patients with various stages of peripheral arterial disease.

## Background

The impact of a disease on a patient's quality of life and level of activities of daily life (ADL) is an important outcome measure in clinical studies [1]. It is well known that perceived quality of life and ADL are significantly impaired in individuals with peripheral arterial disease (PAD) [2-5].

There are several instruments available to measure quality of life in patients with PAD. Both generic instruments, such as the Short-Form 36 (SF36), the Sickness Impact Profile, and the Nottingham Health Profile [6], and disease-specific instruments like the Vascular Quality of Life Questionnaire (VascuQol) and the Claudication Scale (CLAU-S) are frequently used [7,8]. A limitation of these instruments is that they do not focus on level of ADL in terms of self-care, dressing, indoors and outdoors activities, and housekeeping management. Measuring this level of disability is useful, since it is more closely related to impairments and the course of the disease itself. Within the field of PAD, however, there are no instruments available which specifically address the patient's level of ADL.

The AMC (Academic Medical Center) Linear Disability Score (ALDS) is a recently developed generic itembank which measures disability, as expressed by the ability to perform ADL [9,10]. In contrast to the widely used sum score-based questionnaires, the ALDS itembank was developed within the flexible framework of the item-response theory (IRT). The ALDS has already been validated in a large, mixed patient population [11] and in patients suffering from rheumatoid arthritis, stroke and Parkinson's disease [12-14]. The objective of this study was to evaluate the clinimetric properties of the ALDS in patients with different stages of PAD.

## Methods

### Patients

A convenient sample of 62 patients was included in this prospective study. We deliberately selected patients with different stages of disease to evaluate the ALDS for the whole spectrum of PAD. Patients visited the vascular laboratory or vascular nursing ward of our hospital between January 2007 and November 2007. All patients were diagnosed with either intermittent claudication (IC; Rutherford category 1, 2 or 3) or chronic critical limb ischemia (CLI; Rutherford category 4, 5 or 6) [15]. The clinical diagnosis was confirmed by perfusion parameters, such as ankle/brachial index (ABI) and toepressure (TP). Patients were assessed and interviewed by one of the authors (RM). Assessments took place before intervention, consisting of exercise training, revascularization or amputation. The study was approved by the local Institutional Review Board.

### Assessments

We recorded risk factors for atherosclerosis, namely diabetes mellitus, hypertension, smoking, renal failure, hypercholesterolemia, history of coronary artery disease or cerebrovascular disease. In patients with IC, we measured ABI at rest and after exercise. In patients with CLI, we measured ABI at rest and TP.

Quality of life was measured using the VascuQol, which is a sum-score based instrument. The questionnaire consists of 25 items on five domains, i.e. Pain (4 items), Activity (8), Emotional (7), Symptoms (4) and Social (2). Each item is rated as a seven point response scale, with a score of one being the worst and a score of seven the best possible. The total average score is the sum of all 25 items scores divided by 25. For each separate domain an average score can be calculated (sum of all items of one domain divided by the number of items of that domain). So, both the overall score as well as the scores per domain range from one to seven [16]. The VascuQol has shown to be a reliable and valid instrument for assessment of QoL in patients with PAD [7,17].

Disability status was evaluated using the ALDS. For the psychometrical details of IRT in relation to the ALDS, see Additional file 1. The current version of the ALDS itembank consists of 77 items, ranging from very easy (e.g., get out of bed into a chair) to relatively difficult (e.g., walk for more than 15 minutes) [see Additional file 2]. Initially, the ALDS was developed within a dichotomous IRT model with two response options 'I can carry out the activity' and 'I cannot carry out the activity' [9]. However, the dichotomous rating scales were disliked by some respondents as they are perceived as too restrictive. Therefore, the option 'with difficulty' has been added. Currently, each item has three response options, but the response options 'can carry out' and 'can carry out, but with difficulty' are analysed as one response category. In the case that a patient has never performed the activity or answers that he does not know, 'Not applicable' is recorded. The original units of the ALDS scale are (logistic) regression coefficients, expressed in logits. To make the results easier to interpret these scores are linearly transformed into values between 0 and 100. Lower scores represent more disability.

A major strength of an IRT itembank is that researchers, using their clinical judgment, can make their own selections of items from the itembank that are applicable to the population they are investigating. By using a small number of items tailored to the expected ADL level of patients, a detailed clinical picture can be obtained without the need to have all the questions answered by the patient. Even if different sets of items are used for different patient groups, ALDS scores can still be compared because all items are derived from the calibrated itembank. In this way the ALDS can be used to assess patients with a wide range of conditions and levels of functional status.

The methodology [9], the psychometrics of the ALDS in terms of dealing with missing data [18], differences between item measurement characteristics of the itembank in relation to age and sex [19] and the metric properties of ALDS items in mixed types of patient groups [11-14], as well as the statistical power to detect given effect sizes in clinical trials using IRT outcome scales [20] have been examined in depth.

From the ALDS itembank, two questionnaires were composed in this study: one questionnaire for claudicants (29 items), and one questionnaire for patients with critical limb ischemia (27 items). Twenty-three items were in common, covering the whole range of the ALDS itembank. Besides these common items, the claudication questionnaire encompassed six additional, relatively more difficult activities, whereas in the critical limb ischemia questionnaire four extra, relatively easier activities were offered. Selecting a representative range of items is essential to prevent floor and ceiling effects. For example, presenting a slightly disabled patient only items between an ALDS of 10 to 50, the maximum achieved ALDS will be 50 (ceiling effect), whereas with items ranging from 0 through 100, the 'real score' (for example 80) can be achieved. Since the ALDS is based on the IRT, the score is not influenced by the selected items [9]. For the complete ALDS item bank and the selected items in this study, see Additional file 2.

### Clinimetric evaluation

The clinical measurement properties of the ALDS were evaluated in terms of internal consistency reliability, construct validity and clinical validity.

Internal consistency reliability refers to the statistical coherence of the scale items. One measure of internal consistency is the Cronbach's α coefficient, which is based on the (weighted) average correlation of items within a scale [21,22]. Internal consistency is considered to be good if α ≥ 0.80 [23]. We also calculated item-total correlations which represent the correlation of a single item with the sum of all other items. Correlations ≥ 0.40 were conservatively considered to be sufficient.

Construct validity concerns whether the new scale corresponds with other instruments measuring the same health concept and instruments measuring different aspects of health. We assumed that in order for the ALDS to be valid, the ALDS scores had to show a decreasing pattern of associations, with the highest correlation with the disability related Activity domain of the VascuQol, intermediate correlations with the VascuQol subscales Symptom, Pain, Emotional and Social, and the lowest with the impairments in terms of ABI and TP [24,25].

Clinical validity (also known as known-groups validity) refers to the ability of an instrument to discriminate between patient groups with known differences in clinical status. In this study, clinical validity was investigated by comparing the ALDS between patients with IC and patients with CLI, with ALDS scores to be expected higher in patients with IC than in patients with CLI.

The VascuQol was used as benchmark and therefore the analyses focusing the association between functional health and the vascular parameters and the mean score differences between patients with IC and CLI, were also done for the VascuQol and its Activity domain.

### Statistical analysis

Patient characteristics and outcome scores were summarized using descriptive statistics. Distribution of the data was tested with a histogram and the Kolmogorov-Smirnov test. In case of discrepancy between both methods, we regarded the data as not normally distributed. ALDS outcome scores were calculated using a dichotomous IRT model, based on previously published item properties [11] and algorithms implemented in BILOG-MG (version 3.0) and SPSS version 14.0 (SPSS Inc, Chicago, Illinois). In this approach the response options 'can carry out' and 'can carry out, but with difficulty' are analysed as one response category. ALDS items which were rated 'Not applicable' were statistically considered as if they were not presented to that patient [18].

Cronbach's α was obtained using a specific IRT method that allows for missing item responses. The average item-total correlation was calculated using a biserial correlation. Associations between the ALDS (and VascuQol) and other outcome measures were expressed in Pearsons's or Spearman's correlation coefficients, when appropriate. We labelled the strength of the association: correlation coefficients r = 0.00-0.19 were regarded as very weak, r = 0.20-0.39 as weak, r = 0.40-0.59 as moderate, r = 0.60-0.79 as strong and r = 0.80-1.00 as very strong [26]. An unpaired *t*-test was used to compare ALDS and VascuQol scores between the two patients groups. Difference in mean scores between both diagnosis groups was expressed in Cohen's *d *effect size, defined as the difference between the means divided by the pooled standard deviation. An effect size value between 0.50 and 0.80 is considered as a moderate difference, and ≥ 0.80 as substantial [27].

## Results

A total of 62 patients were included, 26 (42%) with intermittent claudication (Rutherford 1 in 6 patients, Rutherford 2 in 13, and Rutherford 3 in 7 patients) and 36 (58%) with critical limb ischemia (Rutherford 4 in 11 patients, Rutherford 5 in 17, and Rutherford 6 in 8 patients). The majority of the patients (71%) were male and the mean age was 68 (± 11) years. Table 1 shows the patient characteristics at time of assessment. The VascuQol Total score, the VascuQol domains Activity, Symptoms, Pain, Emotional and Social, and the ALDS were all normally distributed (histograms showed normal distribution and Kolmogorov-Smirnov test p-values > 0.10). The decrease in ABI, resting ABI, and TP were considered not normally distributed (although the Kolmogorov-Smirnov test had a p-value > 0.05, the histograms did not show a Gaussian distribution).

**Table 1:** Patient characteristics (n = 62) at assessment.

**Characteristics**	**Mean (± sd) or median (range) or n (%)**
Gender, male	44 (71%)

Age	68.4 (± 11.4)

Risk factors	

Diabetes mellitus	22 (35%)

Hypertension	44 (71%)

Current or former smoker	51 (82%)

Renal failure	16 (26%)

Hypercholesterolemia	15 (24%)

History of coronary artery disease	23 (37%)

History of stroke	12 (19%)

Fontaine stage	

II; intermittent claudication	26 (42%)

III or IV; critical limb ischemia	36 (58%)

Definitive treatment	

Conservative	15 (24%)

Endovascular revascularization	36 (58%))

Surgical revascularization	8 (13%)

Amputation	3 (5%)

ABI at rest (in patients with CLI)	0.35 (0-0.59)

Decrease ABI after exercise (in patients with IC)^a^	0.28 (0.09-0.55)

Toe pressure mmHg (in patients with CLI)	19 (0-67)

ALDS	71 (± 17)

VascuQol total	3.7 (± 1.3)

^a^Indicates difference in ABI before and after exercise

The internal consistency reliability of the ALDS in terms of Cronbach's α and item-total correlation turned out to be good; α coefficient > 0.90, average item-total correlation: 0.75.

Table 2 presents the correlations between the ALDS scores and the various subscale scores of the VascuQol. Convergent validity was confirmed with a relatively strong correlation (*r *= 0.64) between the ALDS and the disability related Activity domain of the VascuQol. Moderate correlations were observed between the ALDS and the subscales Symptom (*r *= 0.44) and Social (*r = *0.52), whereas the ALDS was weakly associated with the Emotional and Pain domains (0.30 and 0.28). Table 3 presents the correlations between the ALDS and the VascuQol scores on the one hand and decrease in ABI, resting ABI, and TP. These correlations were (very) weak (*r *range: 0 - 0.38).

**Table 2:** Construct validity; Pearson's correlation coefficients between the ALDS and the VascuQol (n = 62).

	**ALDS**	
VascuQol; *Total score*	r = 0.55	p = < 0.001

VascuQol; domain *Activity*	r = 0.64	p = < 0.001

VascuQol; domain *Symptoms*	r = 0.44	p = < 0.001

VascuQol; domain *Pain*	r = 0.28	p = 0.03

VascuQol; domain *Emotional*	r = 0.30	p = 0.02

VascuQol; domain *Social*	r = 0.52	p = < 0.001

**Table 3:** Construct validity; Spearman correlation coefficients between the ALDS, the VascuQol and clinical indicators (n = 62).

	**ALDS**		**VascuQol** **(Activity)**		**VascuQol** **(Total)**	
Decrease ABI after exercise (in patients with IC)	r = 0.16	p = 0.50	r = 0.00	p = 1.00	r = 0.16	p = 0.50

ABI at rest (in patients with CLI)	r = 0.14	p = 0.49	r = 0.02	p = 0.92	r = 0.20	p = 0.33

Toe pressure (in patients with CLI)	r = 0.19	p = 0.37	r = 0.38	p = 0.06	r = 0.18	p = 0.38

Clinical validity is shown in Table 4. The ALDS score was significantly higher in patients with claudication (ALDS score 80) than in patients with CLI (ALDS score 64). Similar results were obtained for the VascuQol total score (4.5 in patients with claudication and 3.1 in patients with CLI) and the VascuQol domain Activity (4.0 in patients with claudication versus 2.4 in patients with CLI). The effect size values for the ALDS and the VascuQol total and subscale scores were *d *= 0.97, 1.13, and 1.08, respectively.

**Table 4:** Clinical validity: ALDS and VascuQol score of patients with IC (n = 26) and CLI (n = 36).

	**Patient groups**				
	**Intermittent claudication**	**Critical limb ischemia**	**Difference** **(95% confidence interval)**		

ALDS	80 (± 10)	64 (± 18)	16 (8-24)	p < .001^a^	*d *= 0.97

VascuQol (Activity)	4.0 (± 1.6)	2.4 (± 1.1)	1.7 (0.9-2.4)	p < .001^a^	*d *= 1.08

VascuQol (Total)	4.5 (± 1.1)	3.1 (± 1.0)	1.4 (0.9-2.0)	p < .001^a^	*d *= 1.13

^a^Unpaired *t*-test; *d *= Cohen's effect size

## Discussion

In this study, we showed that the ALDS has promising clinical measurement properties to assess the level of disability in patients with PAD. The ALDS demonstrated convincing statistical coherence and was higher in patients with milder disease, who are expected to be less disabled. We could not compare the ALDS with a gold standard, as there is not such an instrument measuring disability available for patients with PAD. A recent study, comparing three questionnaires - two generic questionnaires (the EuroQol and SF-36) and one disease-specific questionnaire (the VascuQol) - showed that the VascuQol is the preferred questionnaire for measuring QoL in patients with PAD [7]. For this reason, we used the VascuQol, and especially its Activity domain, as benchmark for the ALDS analyses. Construct validity was confirmed by a relatively strong association of the ALDS with the domain Activity of the VascuQol, which also measures aspects of physical disability. Construct validity was further supported by decreasing correlations with the other non-disability domains of the VascuQol and the clinical indicators of lower limb ischemia.

The weak correlation between the ALDS (and VascuQol) and clinical indicators of lower limb perfusion in terms of ABI and toe pressure may seem remarkable, but is in line with previous studies in other populations showing that objective disease indicators are not always clearly reflected in (subjective) aspects of functional health [28]. This seems to be true also for patients with PAD. Long et al did not find a correlation between the ABI, the Walking Impairment Questionnaire (WIQ, measuring mobility) and the Physical Component score of the SF36 in patients with symptoms of PAD [24]. Other studies also failed to demonstrate a correlation between the ABI and the SF36 Physical functioning domain and the EuroQol [25,29,30].

The WIQ [31,32] is one of the few instruments that assesses the level of disability in terms of mobility. This questionnaire focuses mainly on walking ability, divided in four subcategories: pain, distance, walking speed and stair climbing. The WIQ has been developed specifically for patients with IC, and does not cover the whole spectrum of PAD. The ALDS carries the advantage that it can be used for both patients with IC and CLI. Moreover, the ALDS focuses on the whole spectrum of basic and complex activities of daily life, including self-care, different mobility levels, housekeeping and outdoor activities.

Most clinicians are used to work with traditional outcome instruments based on sum scores. Although adding up individual item scores to a total score is comprehensibly in use, several problems are associated with this approach. Firstly, all items of the questionnaire have to be presented to patients in order to obtain a summated score. This implies that for a detailed picture of the patient, a long questionnaire encompassing many questions, is needed, increasing patient burden and research effort. This inefficiency has led researchers to shorten health measurement instruments, resulting in less precise scales. Secondly, the ordinal nature of summated scores implies that a given difference in scores at one point on the scale does not necessarily represent the same amount of functional change as an identical difference at another point on the scale. Following growing dissatisfaction with this 'classical' approach, IRT has been introduced to overcome these methodological problems [33].

Measurement instruments based on the IRT have some specific advantages. A clinician can select a set of items which is applicable to the population that is investigated, not all items from the itembank are needed to obtain a score. For example, very easy items do not have to be presented to minor disabled patients. Therefore, the ALDS can be administered in a time-efficient way (in this study between 5-10 minutes). There are some essential aspects to be aware of. As mentioned before, to prevent floor and ceiling effects (i.e. the extent to which respondents score at the bottom or top of a scale) it is very important to ask a patient activities he is able to do and also activities he is not able to do, instead of asking too difficult or too easy questions. If one does so, it does not matter which questions are picked to assess patient's disability level, since the ALDS is based on the IRT. The latter is, as we found out, the most difficult part of the ALDS to appreciate by those who are used to work with the traditional questionnaires.

Some limitations of this study should be recognized. A repeated measurement with an instrument in the same patient or using different interviewers must give more or less the same outcome in the case of an unchanged patient. In the present study, we did not analyze test-retest or between-interviewer reliability. Yet, in a previous study with the ALDS in patients with rheumatoid arthritis, excellent test-retest reliability was found with an Intra Class Coefficient of 0.93 [14]. Other disadvantages are that the ALDS interviewer was not blinded to patient characteristics and that we studied a relatively small number of patients. This must be taking into account when interpreting the results.

As the objective of this validation study was to investigate the measurement properties of ALDS in patients with different stages of PAD, we deliberately selected patients for inclusion, instead of consecutive enrolment, to guarantee that the whole spectrum of PAD was represented in our sample. There is no reason to assume that this non-consecutive inclusion has influenced our psychometrical findings.

## Conclusion

Our study must be seen as a first step in the process of validation of the ALDS in patients with PAD. Further evaluation of this instrument, especially with regard to the test-retest and between-reviewer reliability and the presence of floor and ceiling effects, is needed in a larger consecutive patient population. We think the instrument could be particularly useful in research, to measure the effect of treatment. Before this, the responsiveness of the ALDS to health change over time must be investigated. In conclusion, the results of this pilot study show that the ALDS has promising metric properties and is a potentially useful tool to measure activities of daily life in patients with PAD.

## Abbreviations

ABI: Ankle/brachial index; ADL: Activities of daily life; ALDS: AMC Linear Disability Score; AMC: Academic Medical Center; CLAU-S: Claudication Scale; CLI: Critical limb ischemia; IC: Intermittent claudication; IRT: Item response theory; PAD: Peripheral arterial disease; SD: Standard deviation; SF36: Short-Form 36; TP: Toe pressure; VascuQol: Vascular Quality of Life Questionnaire; WIQ: Walking impairment questionnaire.

## Competing interests

The authors declare that they have no competing interests.

## Authors' contributions

RM has made substantial contributions to design of the study and acquisition and analysis of data, and drafting of the manuscript, JAR has been involved in the design of the study and interpretation of data, as well as in drafting the manuscript, MJWK was involved in interpretation of data and drafting the manuscript, DAL contributed to the design and revised the manuscript critically, RJH was involved in design, analysis and interpretation of the data and drafting of the manuscript. All authors read and approved the final manuscript.

## Supplementary Material

Additional file 1 **Methodology of the ALDS itembank**. Data represent details about the construction of the ALDS itembank. Click here for file

Additional file 2 **ALDS itembank containing 77 items**. Data represent a list of all 77 items of the ALDS itembank, the items we used in our study are marked. Click here for file
