# Supplementary material for: The AMC Linear Disability Score (ALDS): a cross-sectional study with a new generic instrument to measure disability applied to patients with peripheral arterial disease
Source: Health Qual Life Outcomes. 2009 Oct 12;7:88. doi: 10.1186/1477-7525-7-88 (PMC2766362; doi:10.1186/1477-7525-7-88)
Supplement: Additional file 1 — Methodology of the ALDS itembank. Data represent details about the construction of the ALDS itembank. [file 1477-7525-7-88-S1.DOC]

Methodology of the ALDS itembank.

The construction of an item bank based on item response theory (IRT) can be split into four phases: 1) definition of the content; 2) choice of calibration design; 3) data collection; and 4) fitting the ITR model.

*Definition of the content*

The ALDS item bank consists of items measuring the functional status of patients with a broad range of diseases. Functional status was defined as the ability to perform the activities of daily life required to live independently or in an appropriate care setting.[1] Items for inclusion in the ALDS item bank were obtained from a systematic review of generic and disease specific functional health instruments[2] and supplemented by diaries of activities performed by healthy adults. A total of 190 items were identified from over 110 existing functional scales and then described in detail. Two response categories were used: ‘I can carry out the activity’ and ‘I cannot carry out the activity’. If patients had never had the opportunity to experience an activity a ‘not applicable’ response was recorded.[3] Patients were asked, by trained interviewers, whether they could, rather than did, carry out the activities given.

*Choice of calibration design*

The calibration design used in the construction of an item bank describes which items are presented to which patients in the data collection phase. In the ALDS project, data was collected using an incomplete anchored calibration design using 10 items sets, ranging from difficult (set 1) to very easy (set 10). Item set 1 contains activities, which can only be carried out by those who are relatively healthy, and item set 10 those, which can be carried out by all but the most severely disabled patients.[4] Half of all the items in a given item set are common with the item set above and the other half with the item set below, meaning that each item is in two item sets and the whole design is anchored.[5]

*Data collection*

During the development phase of the ALDS, data was collected from over 1000 disabled patients with a broad range of conditions.[6] Patients were interviewed by specially trained nurses and doctors during a visit to one of the vascular surgery, neurology, rheumatology, pulmonology, internal medicine, cardiology, rehabilitation medicine and gerontology outpatients clinics at one general and two teaching hospitals in Amsterdam, the Netherlands. Each patient was presented with one of the item sets in the incomplete anchored calibration design.

*Fitting the ITR model*

The statistical analysis concentrated on the two-parameter logistic IRT model.[7] In this model the probability, *P_ik_*, that patient *k* responds to item *i* in the category 'can carry out' is modeled using

*P_ik_*
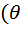
*_ik_*) =
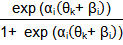

where θ*_k_* denotes the ability of patient *k* to perform activities of daily life. The discrimination parameter (α) and the difficulty parameter (β) describe the measurement characteristics of item *i*. In step (a) items were excluded from further analysis if the item had been presented to fewer than 200 patients or if fewer than 10% or more than 90% of the responses were in the category 'can carry out'. In step (b), the items were examined using the one-parameter logistic IRT model[8] to investigate whether the item difficulty parameter (β*_i_*) was similar for male and female and for younger and older patients. In step (c), estimates of the item parameters (α*_i_* and β*_i_* )were obtained. The fit of the model to the data from each item was assessed using *G*^2^ statistics.[9] Items, for which the fit statistic had a *p*-value of less than 0.01 were excluded from further analysis. In step (d), the dimensionality of the item bank was examined using IRT based full information factor analysis.[10] An exploratory factor analysis was carried out on each of the item sets. To examine the population as a whole, a confirmatory factor analysis was carried out using the data from all 1000 respondents. In addition, Cronbach's coefficient alpha, using a specific IRT method that allows for missing item responses[11], was calculated for each of the item sets (range alpha: 0.92-0.97) and for all of the data (0.98). Steps (a), (b) and (c) were carried out in Bilog[9], using marginal maximum likelihood estimation techniques with an empirically obtained distribution of the person parameters (θ). Step (d) was carried out using TESTFACT.[9]

The resulting and present ALDS item bank contains 77 calibrated items representing a wide range of levels of functional status (see Appendix 2).

*References*

1. Verbrugge LM, Jette AM. The disablement process. Soc Sci Med 1994;38:1–14.
2. Lindeboom R., Vermeulen M, Holman R, de Haan, RJ. Activities of daily living instruments in clinical neurology. Optimizing scales for neurologic assessments. Neurology 2003;60:738–42.
3. Holman R, Glas CAW: Modelling non-ignorable missing data mechanisms with item response theory models. Br J Math Stat Psychol 2005;58:1-17.
4. Holman R, Lindeboom R, Glas CAW, Vermeulen M, de Haan RJ. Constructing an Item Bank Using Item Response Theory: The AMC Linear Disability Score Project. Health Serv Outcomes Res Method 2003;4:19-33.
5. Kolen MJ, Brennan RL. Test equating. Springer, New York, 1995.
6. Holman R, Weisscher N, Glas CAW, Dijkgraaf GW, Vermeulen M, de Haan RJ. The AMC Linear Disability Score item bank: item response theory analysis in a mixed patient population. Health Qual Life Outcomes 2005;3:83.
7. Birnhaum A. Statistical theories of mental test score. Some latent traits models and their use in inferring an examinee’s ability. Addison-Wesley, Reading, MA, 1968.
8. Rasch G: On general laws and the meaning of measurement in psychology. Proceedings of the Fourth Berkely Symposium on Mathematical Statistics and Probability 1961;4:321-34.
9. Du Toit M (editor): IRT from SSI: Bilog-MG, Multilog, Parscale, Testfact. Scientific Software International, Inc, Lincolnwood, IL; 2003.
10. Bock RD, Gibbons RD, Muraki E. Full-information factor analysis. Applied Psychological Measurement 1988;12:261-80.
11. Harvey WR: Estimation of variance and covariance components in the mixed model. Biometrics 1970;26:485-504.
