# Supplementary material for: The AMC Linear Disability Score (ALDS): a cross-sectional study with a new generic instrument to measure disability applied to patients with peripheral arterial disease
Source: Health Qual Life Outcomes. 2009 Oct 12;7:88. doi: 10.1186/1477-7525-7-88 (PMC2766362; doi:10.1186/1477-7525-7-88)
Supplement: Additional file 2 — ALDS itembank containing 77 items. Data represent a list of all 77 items of the ALDS itembank, the items we used in our study are marked. [file 1477-7525-7-88-S2.DOC]

ALDS itembank containing 77 items, hierarchically ranked from difficult to easy, items marked with * were used for patients with intermittent claudication and items marked with # were used for patients with critical limb ischemia.

|  | Item Content |  |  |
| --- | --- | --- | --- |
| Nr | Are you able to ... | Item Difficulty  parameter (β) | Linear transformed ALDS score |
| 1* | Ride a bike for at least 2 hours | –3.05 | 89 |
| 2* ^#^ | Vacuum a flight of stairs | –2.65 | 87 |
| 3* | Carry a bag of shopping upstairs | –2.14 | 85 |
| 4 | Clean a bathroom | –1.96 | 84 |
| 5* ^#^ | Vacuum a room and move light furniture | –1.88 | 84 |
| 6* ^#^ | Fetch groceries for 3–4 days | –1.63 | 82 |
| 7* | Go for a walk in the woods | –1.50 | 81 |
| 8* ^#^ | Travel by local bus or tram | –1.23 | 78 |
| 9* | Walk for more than 15 minutes | –0.82 | 74 |
| 10 | Carry a tray | –0.80 | 74 |
| 11 | Walk up a hill or high bridge | –0.78 | 73 |
| 12* ^#^ | Go shopping for clothes | –0.72 | 73 |
| 13 | Cut your toenails | –0.66 | 72 |
| 14 | Fill in an official form | –0.61 | 71 |
| 15* ^#^ | Go to a party | –0.56 | 70 |
| 16 | Stand for 10 minutes | –0.53 | 70 |
| 17 | Go to a restaurant | –0.48 | 69 |
| 18 | Sweep the floor | –0.45 | 69 |
| 19* ^#^ | Hang and take in a load of washing | –0.44 | 69 |
| 20 | Vacuum without moving any furniture | –0.35 | 67 |
| 21* ^#^ | Move a bed or table | –0.30 | 66 |
| 22 | Use a washing machine | –0.23 | 65 |
| 23 | Reach into a high cupboard | –0.23 | 65 |
| 24* ^#^ | Walk up a flight of stairs | –0.19 | 65 |
| 25* ^#^ | Go to the bank or post office | –0.13 | 64 |
| 26* ^#^ | Walk down a flight of stairs | –0.02 | 62 |
| 27* | Go to the general practitioner | 0.02 | 61 |
| 28 | Use a dustpan and brush | 0.08 | 60 |
| 29* | Go for a short walk (15 min) | 0.07 | 60 |
| 30 | Write a letter | 0.18 | 58 |
| 31 | Change the sheets on a bed | 0.21 | 58 |
| 32* ^#^ | Cross the road | 0.22 | 58 |
| 33 | Open and close a window | 0.24 | 58 |
| 34* ^#^ | Fetch a few things from the shop | 0.29 | 56 |
| 35 | Polish shoes | 0.34 | 56 |
| 36* ^#^ | Have a shower and wash your hair | 0.66 | 50 |
| 37 | Fold up the washing | 0.70 | 50 |
| 38* ^#^ | Dust | 0.70 | 50 |
| 39 | Put on/take off lace-up shoes | 0.76 | 49 |
| 40* ^#^ | Clean a toilet | 0.78 | 48 |
| 41 | Make a bed | 0.84 | 46 |
| 42 | Cut your fingernails | 0.90 | 47 |
| 43 | Reach under a table | 0.91 | 46 |
| 44 | Heat tinned food | 0.92 | 46 |
| 45 | Make eggs or beans on toast | 1.02 | 44 |
| 46 | Reach into a low cupboard | 1.09 | 43 |
| 47* ^#^ | Move between 2 low chairs | 1.14 | 42 |
| 48 | Pick something up from the floor | 1.15 | 42 |
| 49^#^ | Clean a bathroom sink | 1.18 | 42 |
| 50 | Put the washing up away | 1.26 | 40 |
| 51 | Read a newspaper | 1.28 | 40 |
| 52* ^#^ | Get in and out of a car | 1.34 | 39 |
| 53 | Make porridge | 1.37 | 39 |
| 54* ^#^ | Clear the table after a meal | 1.47 | 37 |
| 55 | Peel and core an apple | 1.49 | 37 |
| 56* ^#^ | Prepare breakfast or lunch | 1.52 | 36 |
| 57 | Clean the kitchen surfaces | 1.76 | 32 |
| 58 | Put a chair up to the table | 1.77 | 32 |
| 59 | Eat a meal at the table | 1.79 | 32 |
| 60^#^ | Wash up | 1.86 | 31 |
| 61 | Put on/take off socks and slip on shoes | 1.93 | 30 |
| 62 | Sit up (from lying) in bed | 1.95 | 30 |
| 63 | Get a book off the shelf | 2.11 | 28 |
| 64 | Answer the telephone | 2.15 | 27 |
| 65 | Hang clothes up in a cupboard | 2.19 | 27 |
| 66* ^#^ | Make coffee or tea | 2.35 | 25 |
| 67^#^ | Put long trousers on | 2.38 | 25 |
| 68 | Make a bowl of cereal | 2.28 | 24 |
| 69* ^#^ | Sit on the edge of a bed from lying down | 2.67 | 21 |
| 70 | Move between 2 dining chairs | 2.72 | 20 |
| 71 | Wash and dry your lower body | 2.78 | 20 |
| 72 | Put on/take off a coat | 2.86 | 19 |
| 73 | Wash/dry your face and hands | 2.97 | 18 |
| 74^#^ | Get out of bed into a chair | 2.99 | 18 |
| 75* ^#^ | Go to the toilet | 3.08 | 17 |
| 76 | Wash your lower body (at sink) | 3.24 | 15 |
| 77 | Put on and take off a T-shirt | 3.49 | 11 |
